# Unmasking metabolic dependencies in pancreatic cancer: aberrant polyamine synthesis as a promising new therapeutic target

**DOI:** 10.1038/s41392-023-01662-7

**Published:** 2023-10-27

**Authors:** Gerrit Wolters-Eisfeld, Thilo Hackert, Cenap Güngör

**Affiliations:** Department of General, Visceral and Thoracic Surgery, University Medical Center Hamburg-Eppendorf, Hamburg, Germany

**Keywords:** Gastrointestinal cancer, Translational research

In a recently published *Nature* article, Lee et al. investigated distinct metabolic dependencies of pancreatic ductal adenocarcinoma (PDA) cells compared to normal tissue, and discovered that PDA cells exhibited a unique reliance on de novo ornithine synthesis from glutamine, rather than arginine. This aberrant polyamine synthesis offers a promising therapeutic target for pancreatic cancer.^1^

PDA, often referred to as the “silent killer,” is a deadly disease that often shows no symptoms until it has already spread to other organs. Current diagnostic tools and (chemo)therapies have not been effective in improving patient outcomes with advanced/metastatic PDA, a genetic disease commonly caused by oncogenic *KRAS* mutations, G12D (~50%) and G12V (~30%), respectively. In fact, *KRAS* mutations are present in almost all PDA, considering this neoplasia as the most RAS-addicted among all solid cancers.

Once *KRAS* is mutated, it is “locked” in its active (GTP-bound) state and triggers downstream signaling such as RAF/MEK/ERK and PI3K/AKT/mTOR pathways. This allows oncogenic KRAS to unleash mitogenic processes and develop chemoresistance. There is also significant experimental evidence in PDA supporting the role of oncogenic KRAS signaling in metabolic reprogramming of bioenergetic pathways, specifically involving glutamine and glucose. This metabolic reprogramming is a reversible transcriptional-process influenced by nutrient availability in the tumor microenvironment (TME).^2^ The TME of PDA is characterized by a highly desmoplastic stroma and abnormal vasculature, which restricts nutrient access. Thus, metabolic adaptation and survival in this hostile environment are critical for PDA aggressiveness.

Using metabolic tracing with ^15^N(amine)-glutamine or ^15^N_4_-arginine, Lee and colleagues demonstrated that glutamine was the main source of ornithine and its polyamine derivatives in PDA cells. In contrast, arginine was the preferred source for polyamine synthesis in non-PDA cancers (lung, prostate, breast, colon). Polyamines are essential, low-mass, highly charged molecules that play critical roles in cellular processes related to growth, survival, chromatin structure stabilization, protein and nucleic acid synthesis, and apoptosis in both prokaryotes and eukaryotes. Depleting polyamines leads to cytostasis, emphasizing their importance for normal cell function and growth in mammals.^3^ In an inducible *KRAS*(G12D) mouse model of PDA, the researchers confirmed the preference for glutamine metabolism for in-vivo polyamine synthesis, compared to non-tumor-bearing KRAS wildtype control mice. The authors proposed that the unique metabolic rewiring in PDA cells toward glutamine was a consequence of the low-arginine present in TME, which resulted from arginine breakdown by ARG1-expressing myeloid cells. However, it remains unclear how PDA cells maintain this preferential rewiring for glutamine despite the availability of abundant arginine in the culture media in-vitro.

To understand potential compensatory mechanisms in PDA, the authors conducted gene knockdown studies in AsPC-1 cells targeting the three enzymes involved in ornithine synthesis: *OAT*, *ARG2*, and *GATM* (*glycine amidinotransferase*), as well as the rate-limiting enzyme for polyamine synthesis, *ornithine decarboxylase 1* (ODC1).^4^ Interestingly, silencing *OAT*, but not *ARG2* or *GATM*, significantly reduced glutamine-derived ornithine synthesis and putrescine production. Loss of *OAT* mimicked the effect of *ODC1* knockdown in suppressing AsPC-1 cell-proliferation, which could be rescued by adding putrescine. These results were validated in *KRAS*(G12D) mouse cells and human orthotopic xenografts, where *OAT* loss led to a significant reduction in tumor size. Moreover, since polyamines in the TME were primarily derived from PDA cells rather than non-cancer cells, compensatory uptake following decreased intracellular polyamine levels seemed unlikely.

The study also investigated the role of KRAS in polyamine synthesis and identified KRAS-MEK-KLF6 axis that regulates the transcriptional upregulation of polyamine synthesis genes. Inhibition of KRAS or MEK reduced ornithine and putrescine levels, while silencing *KLF6* decreased expression of polyamine synthesis genes, highlighting the importance of this pathway in PDA.

Comparing the effects of inhibiting OAT and ornithine decarboxylase 1 (ODC1), the rate-limiting enzyme in polyamine synthesis, the authors found that OAT inhibition using 5-fluoromethylornithine (5-FMO) suppressed glutamine-derived ornithine and putrescine synthesis specifically in PDA cells, leading to reduced tumor-growth. In contrast, ODC1 inhibition using difluoromethylornithine (DFMO) primarily affected putrescine synthesis, but had off-target effects on both cancer and non-cancer cells. Combining 5-FMO with a polyamine transport inhibitor showed an additive growth-suppressive effect in PDA cells. In an in-vivo setting, OAT inhibition with 5-FMO effectively suppressed PDA growth without observable toxicity-related changes.

The study also examined the impact of altered polyamine levels on TME and immune cell populations. Decreased polyamine levels resulted in smaller tumors and reduced immunosuppressive granulocytic myeloid-derived suppressor cells. However, polyamine reduction did not significantly affect immune cell populations in tumors or tumor-draining lymph nodes.

Further analysis of the transcriptome and chromatin landscape of PDA cells following *OAT* or *ODC1* knockdown revealed significant changes in gene expression and chromatin accessibility associated with suppressed tumor-growth. These findings highlight the critical role of OAT-mediated polyamine synthesis and suggest it as a potential target for therapeutic intervention in PDA.

Finally, the authors explored the impact of polyamines on the transcriptome and chromatin landscape of PDA using RNA- and ATAC-sequencing.^5^ Silencing *OAT* or *ODC1* resulted in more differentially expressed genes, compared to *ARG2* knockdown and unsupervised clustering showed that silencing *OAT* had greater transcriptional similarity to *ODC1* knockdown. Silencing *OAT* and *ODC1* also led to changes in chromatin accessibility, indicating the involvement of OAT in polyamine-induced alterations. Gene-set enrichment analysis revealed pathways associated with suppressed tumor-growth.

**Fig. 1:**
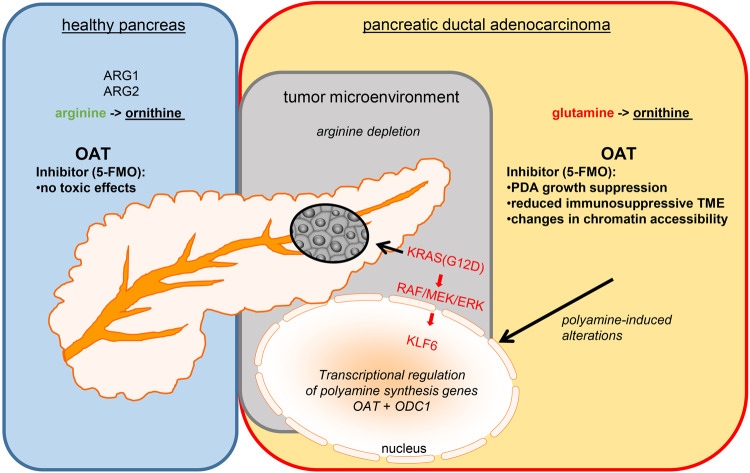
In healthy adults, ornithine is typically produced from arginine through arginase enzymes ARG1 and ARG2 (blue panel). However, the limited availability of arginine in the PDA tumor microenvironment (gray panel) leads to a metabolic shift favoring the unconventional use of glutamine for polyamine synthesis. The KRAS-MEK-KLF6 axis controls the increased expression of genes involved in polyamine synthesis, and inhibiting KRAS or MEK decreases ornithine and putrescine levels. Furthermore, silencing KLF6 reduces the expression of polyamine synthesis genes. In PDA cells, inhibiting OAT with 5-fluoromethylornithine (5-FMO) specifically suppresses the synthesis of ornithine and putrescine derived from glutamine, resulting in reduced tumor growth (orange panel)

Altogether, these findings support the importance of OAT-mediated polyamine synthesis in PDA (Fig. 1) and open up a new therapeutic window for this rebellious and intractable disease.
